# A hybrid bio-inspired sandwich structures for high strain rate energy absorption applications

**DOI:** 10.1038/s41598-024-53521-2

**Published:** 2024-02-04

**Authors:** Jaafar Ghanbari, Pezhman N. Panirani

**Affiliations:** Mechanical Engineering Department, Qom University of Technology, Qom, Iran

**Keywords:** Mechanical engineering, Bioinspired materials

## Abstract

Due to its advantages in terms of enhancing the performance of structures in the desired applications, the bio-inspired design approach has recently attracted the interest of researchers in a number of engineering disciplines. A hybrid bio-inspired design is suggested for the sandwich structures to absorb the energy of the blast loads in the current study. The sandwich structure's core, which often has a regular grid pattern resembling a honeycomb structure, is crucial to how well the panel absorbs energy. In order to achieve the best results, we first chose the structure of the core grid by taking into account potential 2D grids (polygons and multi-pointed stars) through Genetic Algorithm optimization. Next, we combined a bio-inspired bi-tubular thin-walled structure with the core grid to take advantage of its high energy absorption capacity. Finally, the performance of the suggested design is compared with four frequently implemented ones. The results show that the hybrid design has better energy absorption characteristics compared with the bionic and conventional designs presented in the literature.

## Introduction

Due to their great energy absorption capacities, sandwich panels constitute one of the most crucial structures taken into account in impact or blast energy absorption applications. In fields of engineering including civil, transportation, and aerospace, there is a growing need for improved energy-to-weight ratios in order to produce lightweight structures. In the automobile industry, energy absorbers are often column-like structures made to absorb impact energy as much as possible to reduce the energy communicated to the occupants of the car. Sun et al.^1^ investigated the energy absorption characteristics of the variable thickness thin-walled structures. In their study, the thickness of the plates is varied axially and laterally, and their crashworthiness is studied using numerical and experimental techniques. In a similar study, Pang et al.^2^ studied the energy absorption of variable-thickness thin-walled structures under out-of-plane loadings. Xu et al.^3^ reviewed the application of the functionally graded materials (FGMs) for energy absorption applications.

When the impactor's size, shape, and impact position are uncertain, sandwich structures with various core designs are typically utilized to absorb the impact or blast energy. The Specific Energy Absorption (SEA) of the structures is one of the energy absorption qualities that researchers are attempting to improve by putting forward alternative designs. Tarlochan^4^ conducted a review on the use of sandwich structures for energy absorption purposes. Zhao et al.^5^ investigated the energy absorption behavior of sandwich structures with aluminum foam cores through a combination of numerical simulations and experiments. In their study, the structures were subjected to low-velocity impacts. Liu et al.^6^ explored the energy absorption characteristics of sandwich structures with coconut mesocarp cores and glass/carbon fiber reinforced composite sheets Yazdani Sarvestani et al.^7^ studied the energy absorption characteristics of the sandwich structures with 3D printed meta-structure cores. Lian et al.^8^ examined honeycombs with a re-entrant structure under in-plane impact, exploring the energy absorption characteristics of a hybrid model based on their model's design parameters. Dadoura et al.^9^ focused on aluminum tubes filled with foams, identifying the optimal tube sizes and foam properties for enhanced energy absorption. Tang et al.^10^ investigated the acoustic absorption properties of a metamaterial based on a honeycomb-corrugation hybrid core.

The blast-resistance properties of sandwich constructions are the subject of several experimental research. Experimental research on the dynamic behavior of the sandwich panels' sheets and core material under pressure loadings that simulated blasts was conducted by Nemat-Nasser et al.^11^. To achieve the performance of the sandwich constructions, they suggested using an experimental technique in place of actual blast loading. The dynamic response of sandwich structures used as armor against medium-velocity collisions was studied by Kolopp et al.^12^. According to their findings, an aluminum front plate reinforced with aramid stitched textiles is a good decision when defending against a projectile with an impact velocity of 120 m/s and a mass of 127 g. Tarlochan et al.^13^ conducted experimental investigations on the crashworthiness and blast protection capabilities of sandwich structures with tubular inserts. They also analyzed the failure mechanisms of the structures during axial crushing. Patel and Patel^14^ utilized finite element simulations to study the blast resistance of honeycomb sandwich structures with different core designs. They found that foam-filled honeycomb structures exhibit better resistance to blast loads than the base model. Bohara et al.^15^ employed experimental methods and finite element simulations to examine the blast resistance of sandwich structures with auxetic cores. They concluded that dual-mechanism auxetic cores enhance the energy absorption characteristics of the sandwich structures under blast loads.

The impact behavior of the honeycomb sandwich panels used in airplane cargo sections was investigated by Balci et al.^16^ under single and repeated impact conditions. Under repeated impacts of a spherical specimen, impact damages of the front and back plates of the panels are produced. Leijten et al.^17^ conducted a similar investigation for foam core sandwich panels of airplanes subjected to low-velocity collisions. Experimental research on the high-velocity impact behavior of sandwich constructions made of foam cores and aluminum plates was conducted by Abbasi and Nia^18^. Carriere and Cherniaev conduct an analysis of the experimental studies of the sandwich satellite constructions under high-velocity impacts^19^.

Numerous numerical analyses have been suggested in the literature to find the optimum design for sandwich structures to achieve their best energy absorption capacities. An improved design for a tubular thin-walled construction with greater energy absorption capacity was put out by Deng and Liu^20^. The optimization of a composite sandwich construction under oblique low-velocity impacts was studied by Chen et al.^21^. To determine the ideal design parameters, they calibrated the damage model experimentally and applied it to the simulations. The response surface approach was utilized by Meng et al.^22^ to optimize the design of a sandwich structure for selective laser melting. Huang et al.^23^'s analysis of the sandwich constructions' ability to withstand impact loads and subsequent recommendation of an ideal design made use of a multi-objective particle optimization technique.

There is still potential to expand the energy absorption capacity of the structures, despite the fact that the aforementioned and other works significantly increased the ability of the standard sandwich structures to absorb energy. To do this, researchers used a fresh approach to the design idea of the structures, drawing inspiration from complex natural structures like those of plants, insects, and mammals. Several studies on bio-inspired structures for energy absorption applications were reviewed by Ha and Lu^24^. Bamboo is one of the bio-structures that inspire engineers because of how rigid it is in the axial direction. Bamboo may be regarded as a bio-FGM material because of the different sizes and configurations of its radial fibers^25^.

Zou et al.^26^ proposed some thin-walled tubular design concepts inspired by bamboo. They obtained the tube's thickness to yield better energy absorption characteristics by using numerical simulation of the tubes under axial crushing. In a similar study, Palombini et al.^27^ investigated axial and lateral loading of the design concepts of tubular structures inspired by bamboo. Zhang et al.^28^ presented thin-walled design concepts inspired by bamboo and beetle forewing and determined their energy absorption characteristics by experimental and numerical techniques. They investigated the effects of geometrical design parameters, such as the diameter and thickness of the tubes, and their interconnects, on the crashworthiness of the structures. Zhang et al.^29^ proposed a hierarchical honeycomb sandwich panel inspired by pomelo peel for energy absorption applications. In their proposed model, a smaller honeycomb substructure is nested in some of the cells of the larger honeycomb structure. They showed by using numerical simulations that their hierarchical model has better energy absorption characteristics under axial crushing loads. Another honeycomb design inspired by bamboo proposed by Ufodike et al.^30^ and studied its in-plane energy absorption capacity. Chen et al.^31^ presented a design inspired by the beetle’s forewing structure in which a tubular stiffener is inserted in the junctions of the honeycomb structure. They experimentally showed that their design has better performance under axial compression. Several other works investigated bio-inspired ideas based on the microstructure of bamboo^32–35^. Qin et al.^36^ investigated the crashworthiness of a bio-inspired hierarchical multicell tubes under axial impact. They showed that the hierarchical designs show better energy absorptions, compared to the ordinary ones. Deng et al.^37–39^ studied the energy absorption characteristics of corrugated tubes in their works.

In a previous work, the authors of this paper investigated the bionic tubular design inspired by bamboo. They proposed the optimal design to give the best performance in absorbing energy under axial crushing loads^40^. The design consists of two co-axial tubes interconnected with 20 radial ribs—as the optimization amongst eight different designs with variable rib type and number resulted. In the current study, we are interested in finding the best design of the core of the sandwich structure, based on the results of the previous research. To this end, first, we determined the best grid structure the core should have to give the best energy absorption characteristics. After that, a hybrid design is proposed by combining the bionic tubular design with the optimum grid structure to get the best of both components. The design is examined under blast loads and the results are compared with the available studies.

## Grid structure of the core of the sandwich panel

### Energy absorption criteria

To assess the structures' performance in energy absorption applications, the criteria defined in the following are commonly used in the literature.

*Initial Peak Crushing Force (IPCF):* if we plot the force–displacement curve for the structure under crushing, the first peak of it, which represents the force responsible for the beginning of the crushing deformation, is called IPCF. If the crushing force remains below the IPCF, the structure exhibits no visible deformation and maintains its rigidity.

*Energy Absorption (EA):* the total energy absorbed by the structure under impact or crushing loads. It can be calculated by integrating the load–displacement curve as,

1 $$ EA = \int\limits_{0}^{{d_{\max } }} {F(x)\;dx} $$

where $F\left(x\right)$ is the crushing load as a function of the displacement $x$ of the point it is applied and ${d}_{max}$ is the maximum effective deformation.

*Specific Energy Absorption (SEA):* The EA is not so good in comparing the performance of the structures for energy absorption applications since the total absorbed energy varies with the mass of the structure. So, the SEA is defined as the absorbed energy per unit mass of the structure as,

2 $$ SEA = \frac{EA}{m} $$

$m$ being the mass of the structure.

### Possible grid structures

The core of the sandwich structures usually consists of a grid structure assembled by thin-walled plates. In this section, first, we examine the performance of the grid's unit cell in absorbing the crushing energy so that the most efficient of them is chosen to construct the grid of the core. Generally, two types of unit cell may be used in these grids, namely, regular polygons and polygrams. In the following, we obtained the energy absorption characteristics of these types of unit cells with a different number of sides. The material is assumed to be super-austenitic stainless-steel AL-6XN so that we can compare our results with the experimental results of Dharmasena et al.^41^. Since we want to study the energy absorption behavior of the structures under blast loadings, the rate-dependent Johnson–Cook constitutive model is employed throughout this study,

3 $$ \sigma = \left[ {A + B \times (\varepsilon_{pl} )^{n} } \right] \times \left[ {1 + C \times ln\left( {\frac{{\varepsilon_{pl} }}{{\varepsilon_{0} }}} \right)} \right] \times \left( {1 - \widehat{\theta }^{m} } \right) $$

In the above, ${\varepsilon }_{pl}$ is the plastic strain, $\dot{{\varepsilon }_{pl}}$ is the strain rate, and $\theta$ is the temperature. The constants A, B, C, n, and m are listed in Table 1. It should be noted that the parameters of the Johnson–Cook model are obtained through experiments and are dependent on the specific experimental conditions used. Typically, the split-Hopkinson pressure bar experiment is used to generate stress–strain curves of specimens at specific strain rates. Changing the strain rate results in a new set of parameters for the model. However, since materials under blast loads can experience a wide range of strain rates, the numerical values of the parameters may not be suitable for such cases. Therefore, a calibration study is necessary to obtain a set of parameters that best match the experimental data. In this study, the NSGA-2 algorithm is used to calibrate the parameters so that the results of the simulations match those of the experimental data, which will be discussed in the following section.

**Table 1:** Mechanical properties and Johnson–Cook parameters for the Al-6XN stainless steel.

Material property	Original from Ref.^42^	Calibrated
Density (kg/m^3^)	7850	7850
Young’s modulus (GPa)	196	161
Poisson’s ratio	0.35	0.35
A (MPa)	400	400
B (MPa)	1500	871.15
n	0.4	0.1241
Reference strain rate	0.001	0.001
C	0.04	0.055
Ref. temperature (K)	293	293
Melting temperature (K)	1800	1800
m	1.2	1.2

As the grid structures we are covering in this study are made by thin-walled crossing plates forming an array of regular polygons or polyrams, their building blocks, i.e., the polygons or polyrams are studied under crushing loads so that the best crushing energy-absorbing polygon is determined. To this end, different type of polygons with different number of sides (as listed in Table 2) are simulated separately (isolated, not in the array of a core grid structure). To eliminate the size effect of the polygons, all the cells are circumscribed by a cylinder with a 50 mm radius and 100 mm height. All cells are assumed to have a height of 100 mm, and a crushing load is applied until 75% of their original length, i.e., 75 mm is compressed. Consequently, ${d}_{max}$ is equal to 75 mm throughout the analyses.

**Table 2:** Energy absorption characteristics of the regular polygons.

Number of sides	Shell thickness (mm)	EA (kJ)	SEA (kJ/kg)	Mass (kg)
3	2.945	62.532	**104.22**	0.6
4	2.705	56.232	93.72	0.6
5	2.605	53.358	88.93	0.6
6	2.55	50.742	84.57	0.6
7	2.52	48.336	80.56	0.6
8	2.5	47.088	78.48	0.6
9	2.485	47.094	78.49	0.6
10	2.475	48.282	80.47	0.6
11	2.47	47.346	78.91	0.6
12	2.465	46.386	77.31	0.6
13	2.46	45.684	76.14	0.6
14	2.457	45.144	75.24	0.6
15	2.455	44.79	74.65	0.6
16	2.452	44.376	73.96	0.6
17	2.451	43.926	73.21	0.6
18	2.45	43.44	72.40	0.6
19	2.448	42.906	71.51	0.6
20	2.446	42.81	71.35	0.6

Significant values are in bold.

**Figure 1:**
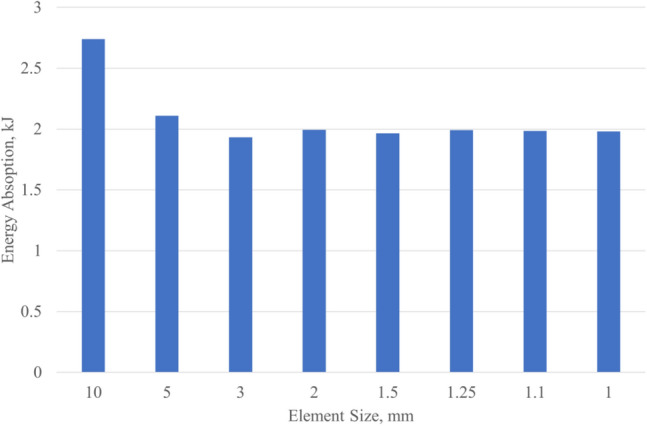
Energy absorption of a bi-tube for different element sizes.

To obtain the suitable element size for the crushing simulations, a mesh convergence study is performed as follows. A bi-tube with the geometrical dimensions mentioned above is constructed and meshed with variable element sizes. After crushing it up to 75 mm compression, its EA is calculated which are shown in Fig. 1. It follows that the energy absorption of the tube remained relatively constant for element sizes smaller than 1.1 mm. Therefore, a 1 mm size was chosen for all subsequent simulations, ensuring an accurate representation of the crushing behavior without unnecessarily increasing computational demands. We have incorporated additional clarifications within the manuscript to address this point as well.

After FEM analysis is completed, we can obtain the reaction force of the crushing (displacement-control) load, and the integration in Eq. (1) is performed numerically. To exclude other factors, the thickness of the plates in these unit cells is chosen in a way that their weight kept constant. The top plate is compressed by a velocity equal to 100 m/s. A 4-node doubly curved shell with reduced integration (S4R) element with an edge size equal to 1 mm is used throughout the simulations. For the crushing simulations described in “Bio-inspired core design”, the thin-walled models are positioned between two rigid anvils. The lower anvil is completely fixed, while the upper one moves downward at a constant velocity of 100 m/s. All degrees of freedom except the axial movement are constrained for the upper anvil. The thin-walled structures themselves are only subjected to a general contact constraint with the anvils, without any additional explicit constraints.

Both the face sheets and the core structure are modeled using the same element type and size. The materials for the face sheets are chosen to be the same as the core structure. The proper element size (1 mm) is obtained by the mesh-size convergence study with the total absorbed energy as the convergence criteria. The results for the unit cells with the regular polygons with different number of sides are listed in Table 2.

Initial and deformed shapes of polygons with 3, 6, and 12 sides are shown in Fig. 4. It follows from the results of Table 2 that the triangular unit cell provides the best SEA amongst the regular polygons. By increasing the number of sides in the polygons, one can observe that the SEA asymptotically approaches a constant value.

**Figure 2:**
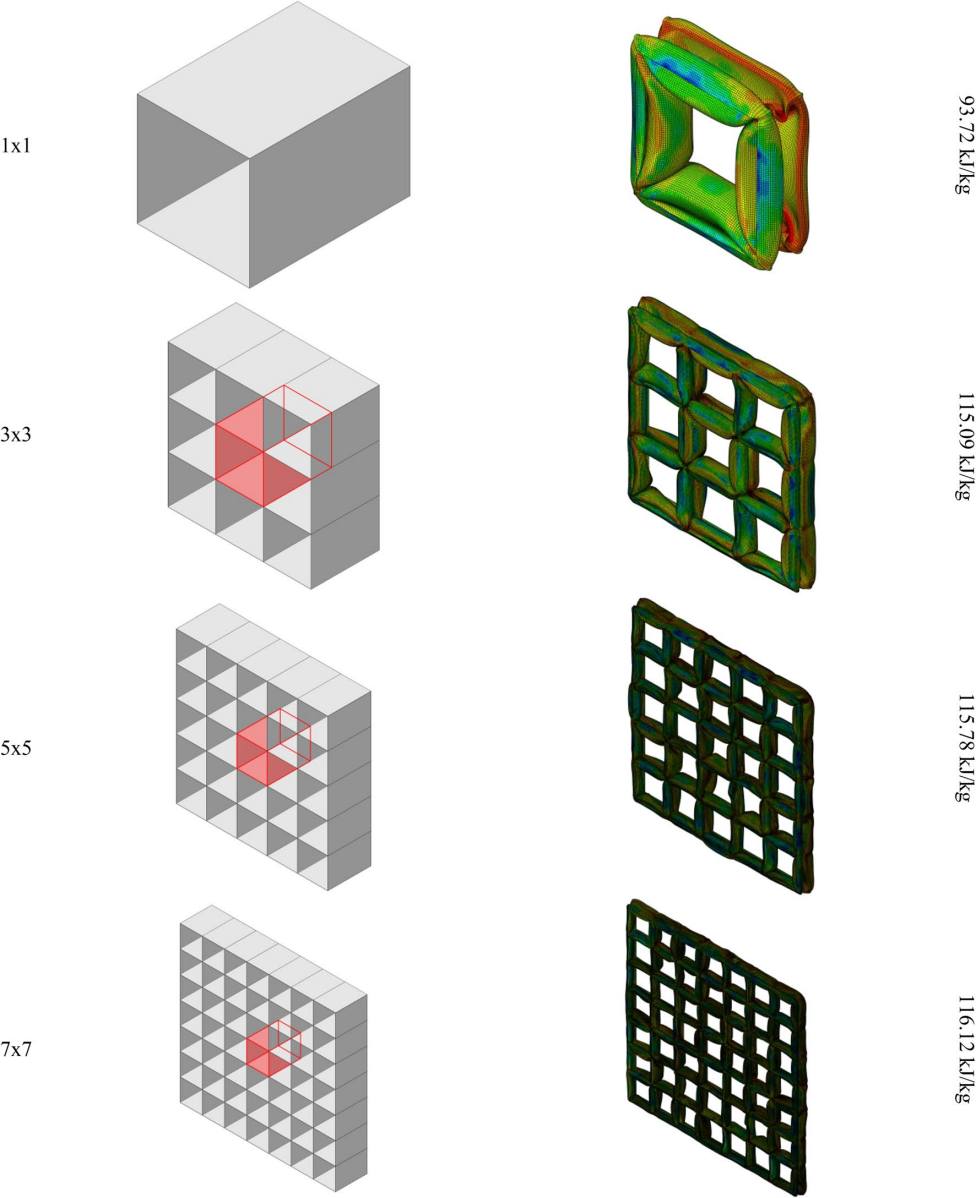
Rectangular grids with different number of cell layers surrounding the unit cell. The SEA is calculated for the highlighted cell.

The results for the unit cells with the regular polygrams are listed in Table 3. Although the behavior of the polygrams is not similar to the polygons—as it fluctuates with the increased number of vertices, the pentagram shows the best SEA amongst the polygrams. Figure 5 illustrates the deformation of the polygrams with 6, 8, and 12 vertices (Fig. 2).

**Table 3:** Energy absorption characteristics of the regular polyrams.

Number of vertices	Shell thickness (mm)	EA (kJ)	SEA (kJ/kg)	Mass (kg)
5	1.608	66.93	**111.55**	0.6
6	1.472	62.004	103.34	0.6
7	1.12	63.816	106.36	0.6
8	1.036	59.796	99.66	0.6
9	0.864	60.882	101.47	0.6
10	0.804	60.084	100.14	0.6
11	0.702	56.61	94.35	0.6
12	0.66	62.55	104.25	0.6

Significant values are in bold.

It is important to note that not all polygons and polygrams are suitable as unit cells for the grid structure of the core, as we are only considering grids that are formed by intersecting parallel vertical plates. Therefore, we only take into account triangular, rectangular, and hexagonal polygons, as well as the hexagram. Additionally, an individual unit cell cannot accurately represent the performance of the grid structure because the deformation of each cell can be affected by its neighboring cells, which may share some edges and/or vertices. In order to determine the SEA of a single cell, it is necessary to consider how many surrounding layers are required. It has been determined that, when studying a grid structure, only one surrounding layer is adequate for determining the deformation of a single cell. This is because the deformation of the cell while connected to other cells in the grid structure is fully defined, and other cells that are not directly connected to the one being studied do not affect its deformation or SEA properties. For cells encompassed in a grid, the cell itself is connected to surrounding cells, and symmetric boundary conditions (constrained in the normal direction to the cutting plane of the boundary and in-plane sliding) are applied to the lateral boundary of the structures.

**Figure 3:**
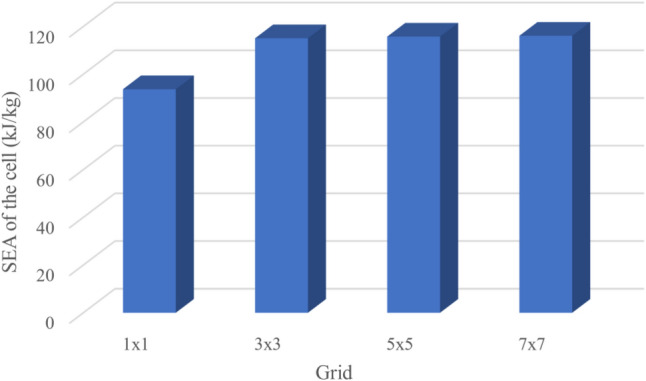
The SEA of the rectangular unit cell for grids with different number of cell layers.

**Figure 4:**
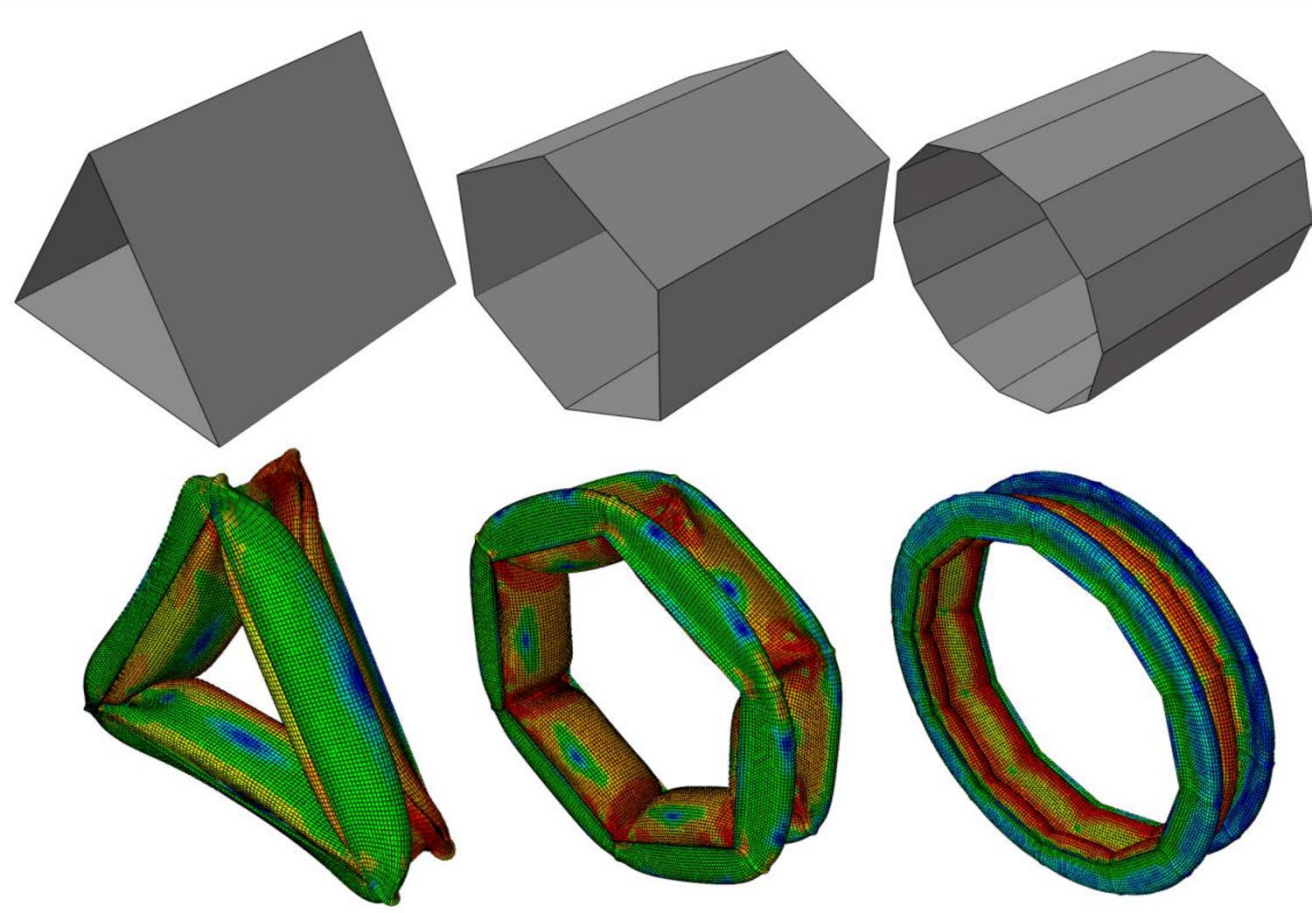
Deformation of the polygons under crushing load.

**Figure 5:**
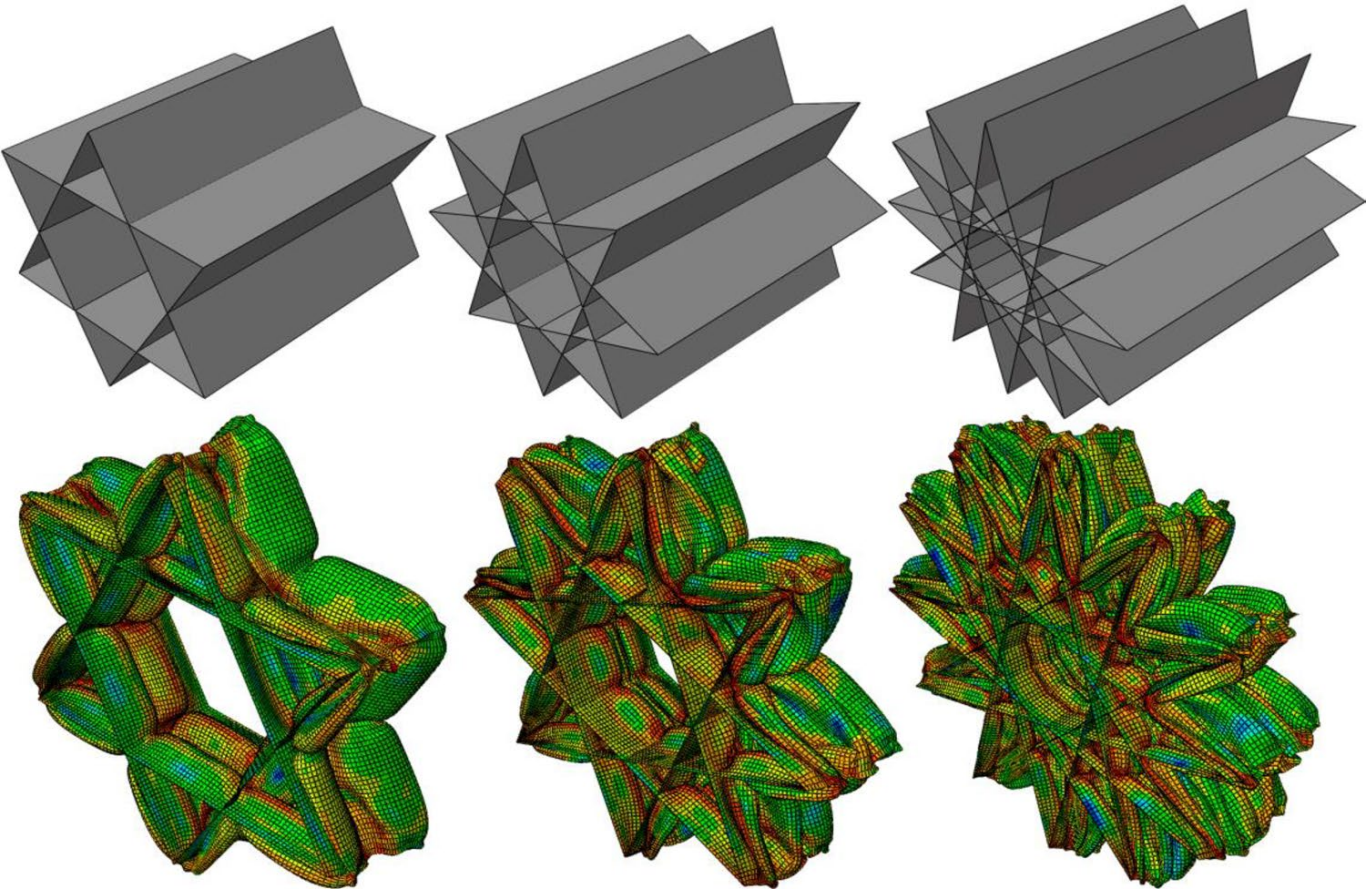
Initial and deformed shapes of the polygrams with 6, 8, and 12 vertices.

Figure 2 illustrates undeformed and post-crushing configurations for rectangular cell grids of varying size, with the cell targeted for SEA quantification highlighted. While overall deformation patterns appear analogous across grid dimensions, derived SEA values in Fig. 2 reveal discernible differences. The solitary cell exhibits lower SEA compared to cells bounded by one or more adjacent cell layers. However, SEA appears to converge based on the number of surrounding cell layers, as increasing from one to three layers yields minimal further variation (Fig. 3). These findings indicate that a single cell layer surrounding the target cell sufficiently replicates the SEA response of an expansive grid structure (Figs. 4, 5).

**Figure 6:**
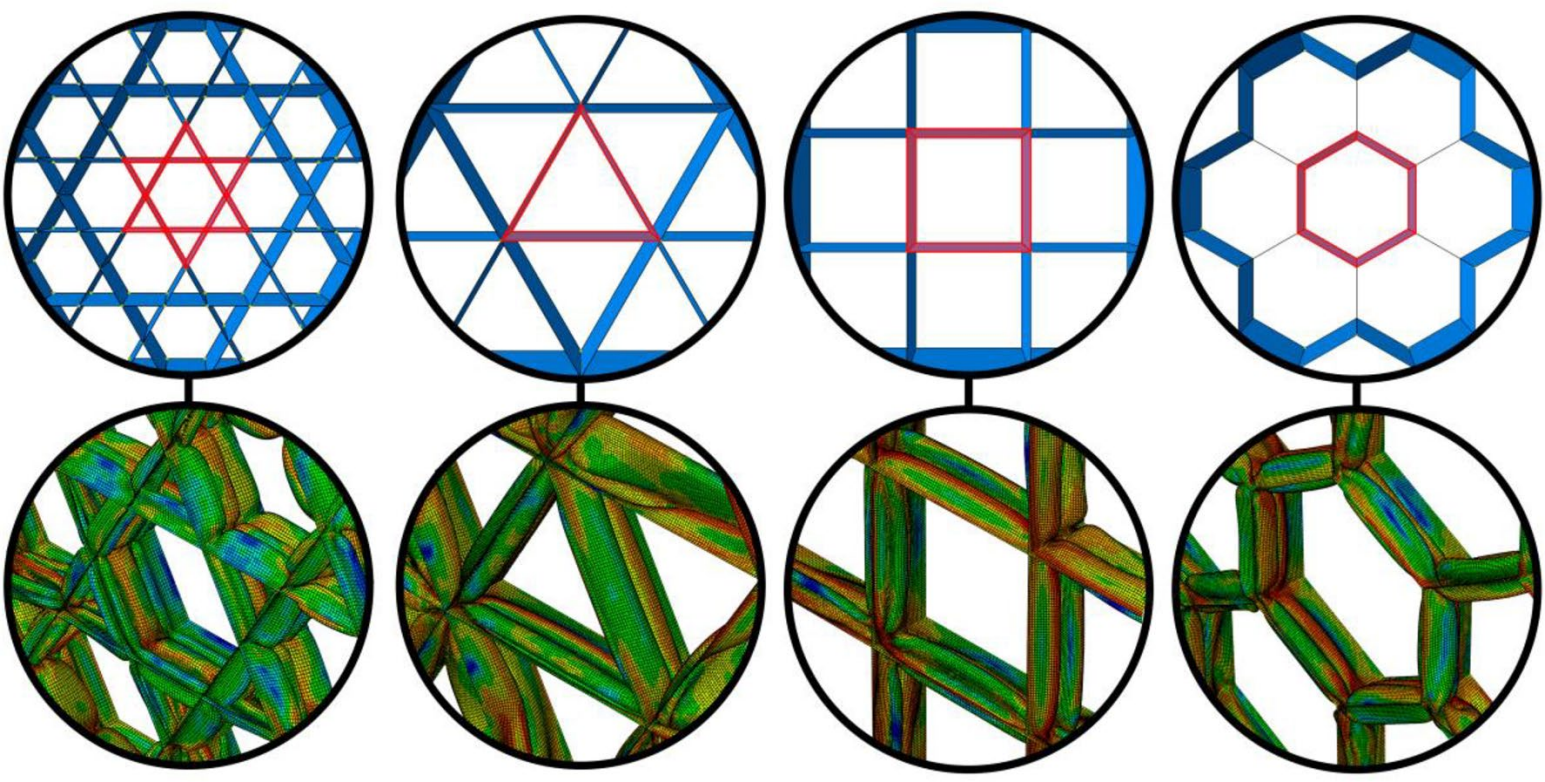
The grid structures with the highlighted unit cell and their deformed state.

Figure 6 illustrates the grids with highlighted unit cells, as well as their deformed states after the crushing deformation has been applied. Note that in the Fig. 6, the perspective view is turned on for better illustration, and the thickness of the plates are all the same.

To ensure accurate computation of energy absorption characteristics, it is important to consider the fact that the rectangular and hexagonal unit cells share their edges with neighboring cells. As a result, when calculating the total mass of the unit cell, only half of the thickness of the plates that are shared with the neighboring cells should be taken into account. Table 4 shows the results for the grid (containing the unit cell surrounded by one neighboring cell) and the unit cell itself. The results show that the triangular unit cell shows better energy absorption characteristics amongst other cell types, and thus, we choose this type in the following to get a hybrid design of the core combining this grid with the bio-inspired tubular structures to further increase the absorbed energy of the sandwich structure.

**Table 4:** The results for the grid structures and their unit cell.

Cell type	Wall thickness (mm)	EA (kJ)	Grid SEA (kJ/kg)	Cell SEA (kJ/kg)	Mass (kg)
Triangular	2.945	85.482	**142.47**	**104.22**	0.6
Rectangular	2.705	69.054	115.09	93.72	0.6
Hexagonal	2.55	74.868	124.78	84.57	0.6
Hexagram	1.472	67.638	112.73	103.34	0.6

Significant values are in bold.

It is noted that the behavior of a polygonal thin-walled structure differs when considered alone or in a structure surrounded by neighbors of the same shape as in the core grid structure. In studying cells, their SEA (Cell SEA) has been observed and ranked in descending order as triangular, hexagram, rectangular, and hexagonal structures. However, when examined as part of a grid structure surrounded by adjacent cells, the SEA of a single polygon (Grid SEA) is obtained in a different order: triangular, hexagonal, rectangular, and hexagram. This discrepancy in ranking can be attributed to the fact that cells behave differently under crushing loads when connected to neighboring cells compared to when they are studied separately. When cells are connected to other cells, their deformation pattern, particularly at the corners where they are joined to neighboring cells, changes, resulting in a change in their SEA.

## Bio-inspired core design

### Core design inspired by the beetle forewing

**Figure 7:**
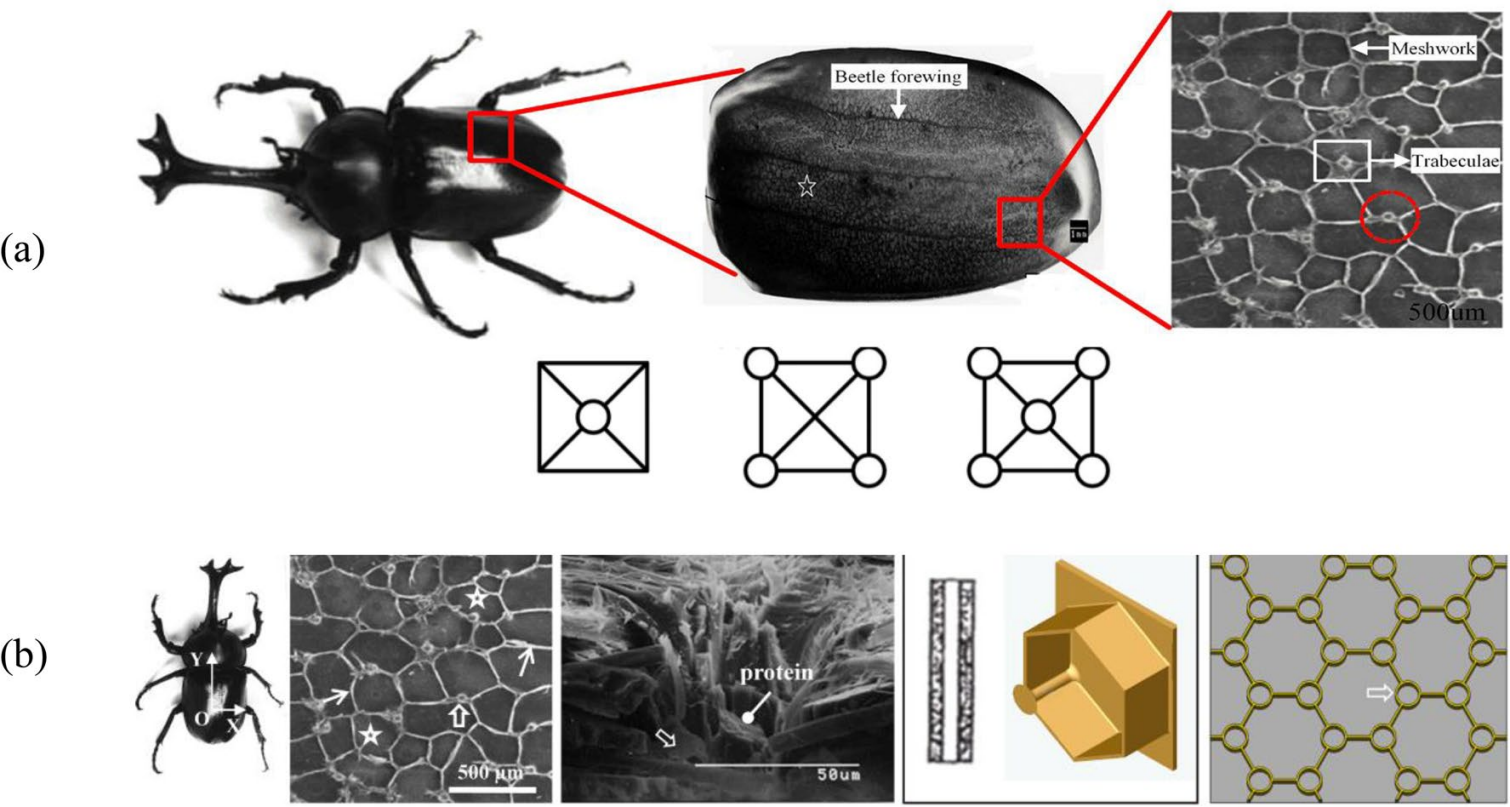
(**a**) The beetle's forewing structure, and the proposed bionic designs of Ref.^43^, (**b**) Beetle-inspired design of Ref.^44^.

Beetle's exoskeleton has exceptional mechanical characteristics as it can withstand large forces and drops from high places, and so, become researchers' interest to study its microstructure to engage in engineering applications. The beetle forewing microstructure consists of a grid structure mixed with some cylindrical trabeculae (Fig. 7).

Zhang et al.^43^ proposed some design concepts of thin-walled structures inspired by the beetle forewing, which are illustrated in Fig. 7-a. They studied the crashworthiness of the proposed designs using the FE simulations and design optimization of the designs with better energy absorption characteristics under crushing loads. They concluded that the bionic designs show better SEA compared with the traditional structures.

Chen et al.^44^ studied a honeycomb grid combined with tubular structures at the vertices as an inspiration for the beetle's forewing under compression (Fig. 7-b). They examined the horizontal as well as the vertical configurations of the tubes with both numerical and experimental techniques.

**Figure 8:**
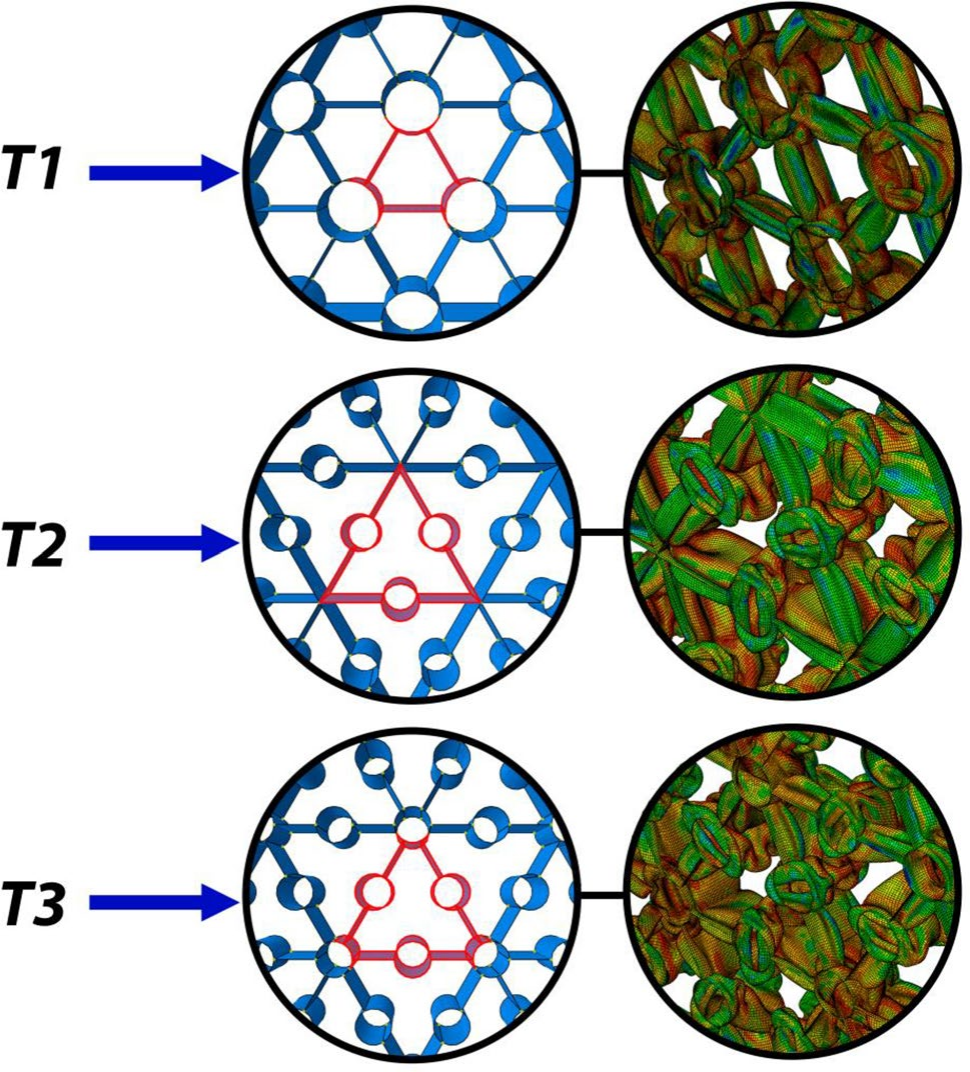
Designs inspired by the beetle's forewing and their deformed state after the crushing load is applied.

Inspired by the beetle's forewing and bearing in mind the design concepts of the studies as mentioned above, three cases are the candidates for the bionic design of the triangular grid structure. These cases are depicted in Fig. 8 as T1, T2, and T3 designs for the reinforcing tubes located at the vertices, edges, and both locations, respectively. The corresponding unit cells are highlighted in the figure. As before, to calculate the energy absorption characteristics, at least one row of the cells surrounds the unit cell to include the effects of the shared vertices and edges on the deformation and energy absorption behavior of the grid. All models are kept at equal mass by calculating the thickness of the walls. To obtain the best possible design parameters (the radius of the tubes) of these design concepts, the Neighborhood Cultivation Genetic Algorithm (NCGA) is employed in the Dassault Systèmes Simulia Isight software with the optimization parameters listed in Table 5 to optimize the designs. Within the optimization node of the Isight workflow, we tailored the configuration of the optimization algorithm and its associated parameters. We selected the NCGA algorithm for its efficacy in this context, employing a population size of 6 and 12 generations. The remaining options were maintained at their default settings. This choice of population and generation numbers was guided by a critical objective: to practically manage computational costs. This optimization process necessitates the execution of 72 full-model simulations, each simulating a comprehensive 75% crushing of the structure's original length. Therefore, striking a balance between thorough exploration and computational efficiency was paramount.

**Table 5:** Optimization technique parameters of the ncga optimization.

Population size	6
Number of generations	12
Crossover type	1
Crossover rate	1.0
Mutation rate	0.01
Gene size	20

The results for the energy absorption characteristics, as well as the radius of the tubes, are shown in Table 6. Comparing the results, it is clear that the design type T1 with the tubes located at the vertices of the triangular design is the best design, with a 28% improvement in the SEA compared to the classical triangular grid structure. While the T1 model appears to produce the best results, the T3 model also shows comparable performance. It is worth noting that both models feature tubular stiffeners at the joints of the vertical plates, which are observed to significantly increase the crushing stiffness of the structure. This may explain why the T3 model performs nearly as well as the T1 model. So, we conclude this section with the fact that the bionic grid design inspired by the beetle's forewing microstructure with improved energy absorption characteristics is the triangular grid structure enhanced by placing tubes at the vertices of the grid.

**Table 6:** The results for the bionic grids.

Design type	Tube radius (mm)	Wall thickness (mm)	EA (kJ)	SEA (kJ/kg)	Mass (kg)
Classical triangular (Fig. 3)	–	2.945	85.482	142.47	0.6
T1	21.06	2.875	109.446	**182.41**	0.6
T2	10.15	1.96	94.194	156.99	0.6
T3	10.15	1.945	107.4	179	0.6

Significant values are in bold.

### Hybrid design inspired by bamboo

**Figure 9:**
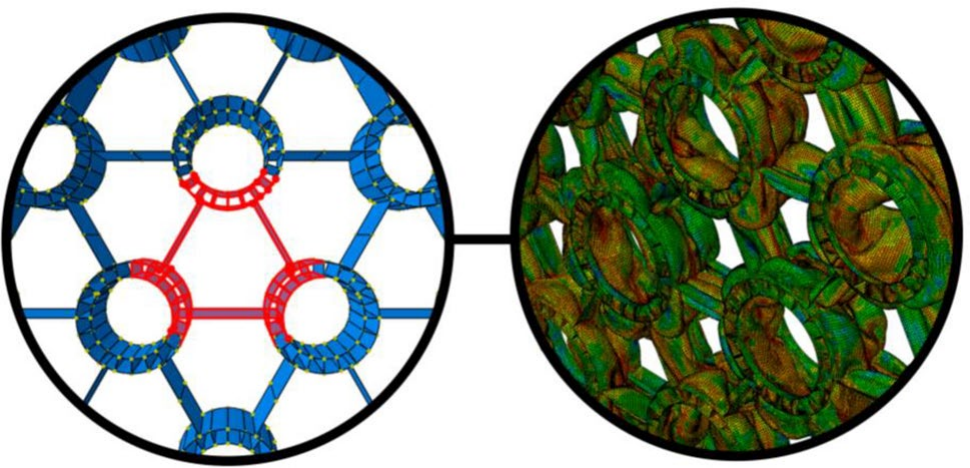
The hybrid bionic design and its deformed state.

As noted before, the microstructure of the bamboo consists of co-axial tubes with some interconnecting ribs between them. The authors have studied some of the bionic thin-walled structures inspired by bamboo and concluded that the bi-tubular design with 20 plain radial interconnects shows better energy absorption characteristics based on optimization algorithms^40^. In this mentioned work, the radii of the tubes and their thickness, as well as the design of their interconnects (as a choice of various proposed options) were the design parameters for the optimization study. Based on these results, we combined the design concept proposed in the previous section with the previous study (with the best SEA for the axial crushing force as the objective of the optimization) to get the new hybrid bionic design for the core grid of the sandwich structure (Fig. 9). To optimize the energy absorption characteristics, another optimization study with the radii of the tubes being the design parameters is performed in the Isight software using the NCGA algorithm with the parameters listed in Table 7.

**Table 7:** Optimization technique parameters for the hybrid bionic design.

Population size	6
Number of generations	12
Crossover type	1
Crossover rate	1.0
Mutation rate	0.01
Gene size	20

The results of the optimized design with the design parameters are listed in Table 8. It follows that the new hybrid design shows a 3.3% improvement compared with the design proposed in the previous section (bionic design inspired by the beetle's forewing) and a 33% increase in the SEA compared with the classical triangular grid core. So, the proposed hybrid bionic design is chosen as the preferred design for the analysis of the sandwich structure under blast loadings.

**Table 8:** Design parameters and energy absorption properties of the optimized hybrid bionic design.

Inner tube radius (mm)	**21.79**
Outer tube radius (mm)	28.82
Wall thickness (mm)	1.402
EA (kJ)	112.88
SEA (kJ/kg)	188.13
Mass (kg)	0.6

Significant values are in bold.

## The sandwich structure under blast loading

### Correlation with the experimental data

**Figure 10:**
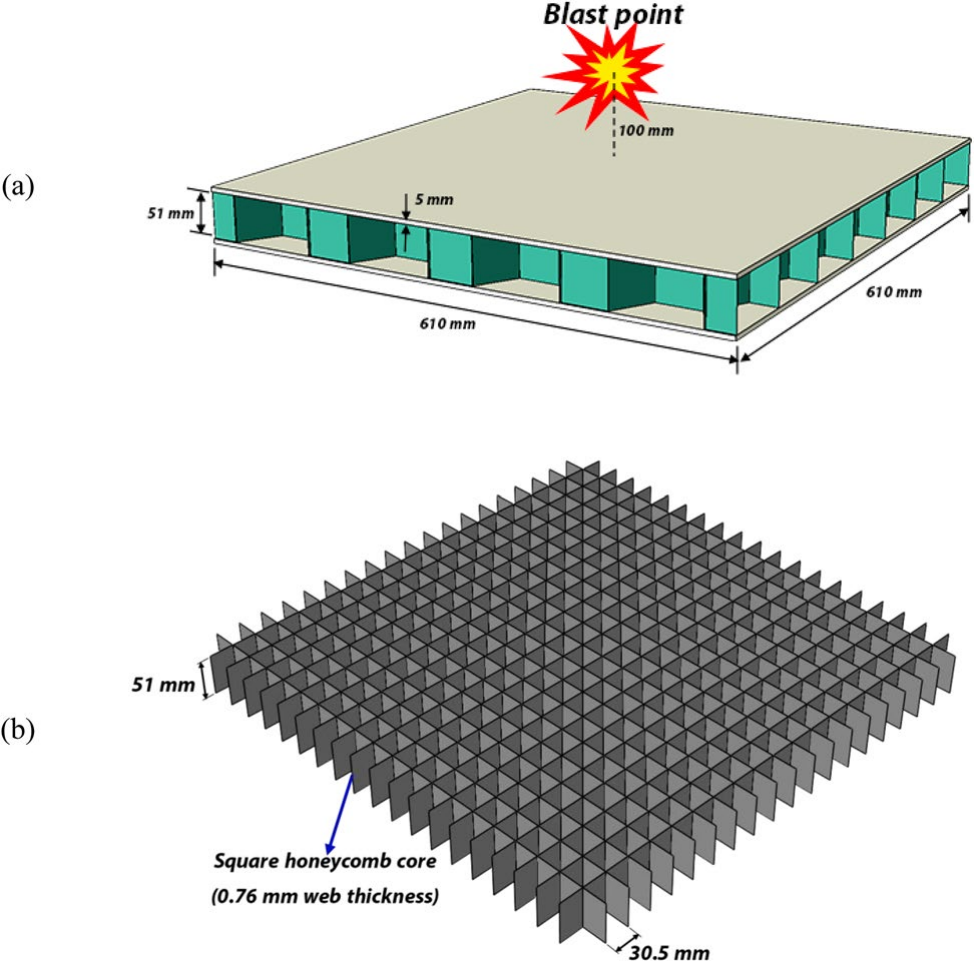
(**a**) The schematic of the blast simulations, (**b**) the core structure.

In this section, we investigate the performance of the proposed hybrid bionic sandwich structure under blast loadings. To do so, first of all, we need to make sure that the simulations are set correctly, and the obtained results represent the actual behavior as closely as possible. Three blast loads corresponding to 1, 2, and 3 kg TNT are modeled using the conwep method in the Abaqus software. The element size is equal to 1 mm as per the mesh convergence study from the previous section. The models are discretized by 4-noded double-curve reduced integration shell (S4R) elements. The sandwich structure consists of 5 mm-thick top and bottom plates with a rectangular grid core structure constructed by 0.76 mm-thick plates. The spacing between the core ribs is 30.5 mm, and its height is equal to 51 mm. The schematic of the models is shown in Fig. 10. Since we want to compare the simulation results with those of the experimental data of Dharmasena et al.^41^, the dimensions and geometry of the sandwich structure are in accordance with the physical model of Ref.^41^. The material is the super-austenitic stainless steel AL-6XN which is modeled using the Johnson–Cook material model with the parameters listed in Table 1.

**Figure 11:**
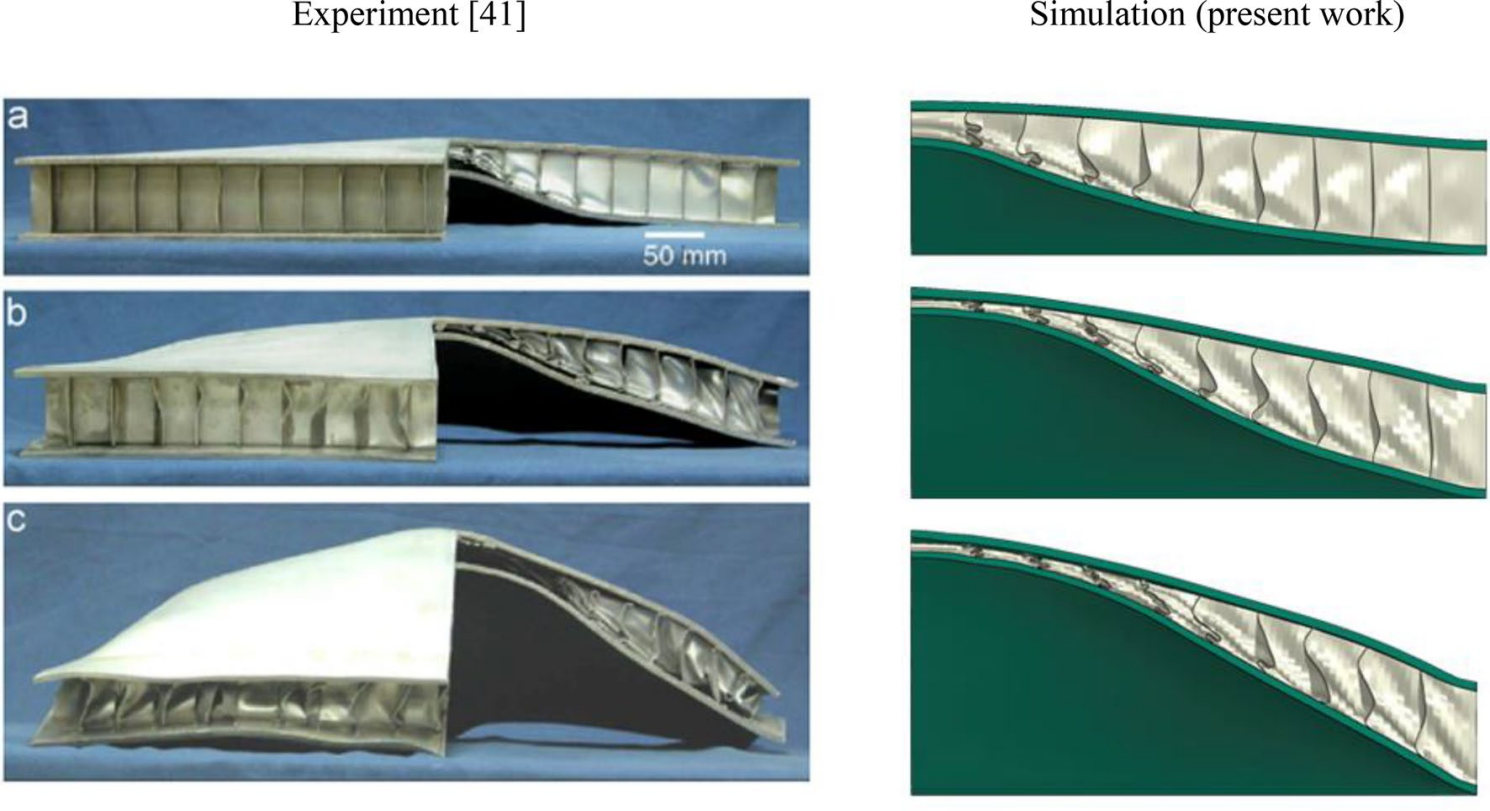
Deformed configuration of the sandwich structure from the simulations in comparison with the experimental results^41^ for (**a**) 1 kg, (**b**) 2 kg, and (**c**) 3 kg TNT equivalent blast loads.

The deformed shape of the structures under the blast loads are depicted in Fig. 11 as well as those of the experimental study of Ref.^41^. As can be seen, there is a good agreement between the simulations and the experimental data. To further tune the simulation parameters, the displacement of the top plate is compared in Table 9. Although the simulation results obtained by using the Johnson–Cook parameters of the Ref.^42^ shows good agreement for the case of 2 kg TNT, but on average of the three cases, there is some 17.6% difference between the experimental data and the simulation results for the maximum deflection of the top plate under the three blast loadings. Discrepancies between simulations and experiments, especially in complex scenarios like high-rate plastic deformation under blast loads, are unsurprising. While the Johnson–Cook model is widely used, it's not perfect for handling such diverse strain rates, particularly over a wide range. Additionally, its coefficients are often determined through specific experimental setups like split Hopkinson pressure bar tests, which operate at certain strain rates. This can lead to the model performing well around those specific rates but faltering when confronted with the broader spectrum encountered in blasts. In this study, the 2 kg TNT blast appears to fall within the range of strain rates used to calibrate the Johnson–Cook model’s coefficients, leading to relatively lower discrepancies compared to other cases. However, to further improve accuracy, we have conducted a multi-objective optimization using the NCGA algorithm, with the experimental deformation of the top plate being the objectives of the three loading cases and the Johnson–Cook parameters being the optimization variables. The results of the calibrated Johnson–Cook parameters are listed in Table 1, with the corresponding simulation results being listed in Table 9. It follows that the calibration reduced the error between the simulation results and those of the experiments, especially in the case of 2 kg TNT. The average error is reduced from 17.66% for the original parameters to 10.76% for the calibrated parameters. So, we confidently use the calibrated Johnson–Cook parameters obtained by the multi-objective optimization for the simulations throughout this study.

**Table 9:** Results for the top plate displacement.

TNT mass	Top plate maximum deformation (mm)				
	Experiment^33^	Simulation with original parameters	Error (%)	Simulation with calibrated parameters	Error (%)
1 kg	47.06	69.50	32.28	58.10	19.00
2 kg	98.82	109.28	9.57	97.98	0.85
3 kg	158.08	140.50	11.12	138.43	12.43
Average error (%)			17.66		10.76

**Figure 12:**
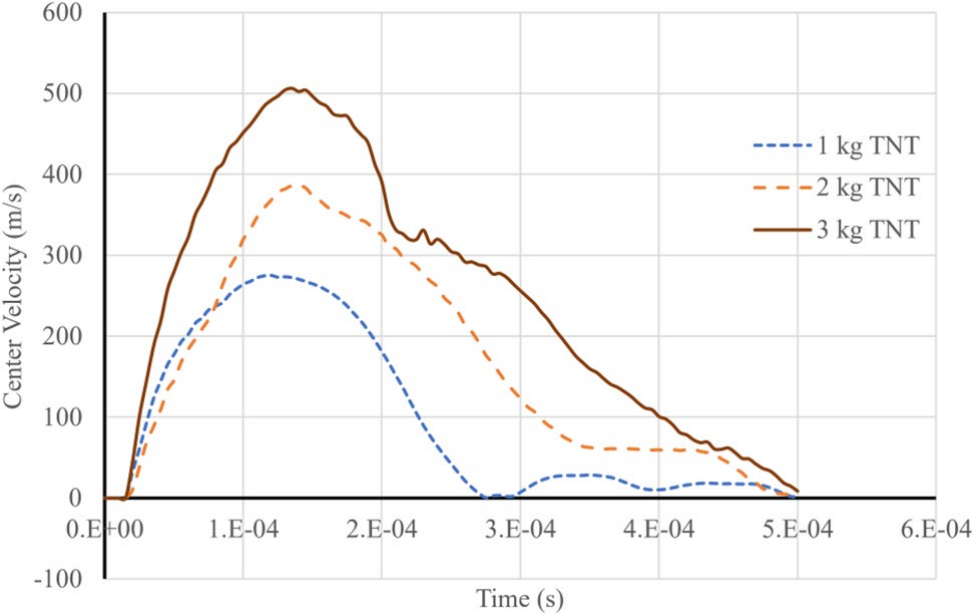
Top sheet center velocity for three blast loads.

Figure 12 presents the velocity of the top sheet's center for three distinct blast loads. Upon examination, it becomes evident that the deformation velocity lies within the order of hundreds of meters per second. This observation substantiates the rationale behind the selection of the crushing speed, as delineated in “Grid structure of the core of the sandwich panel” of the manuscript. The choice of crushing speed is crucial, as it directly impacts the accuracy and relevance of the analysis conducted in this study. By selecting a crushing speed that corresponds to the experimentally observed deformation velocities, the authors ensured that the simulation results would appropriately reflect the actual behavior of the materials and structures under blast loads. Consequently, this choice lends greater credibility and validity to the conclusions drawn from the research.

**Figure 13:**
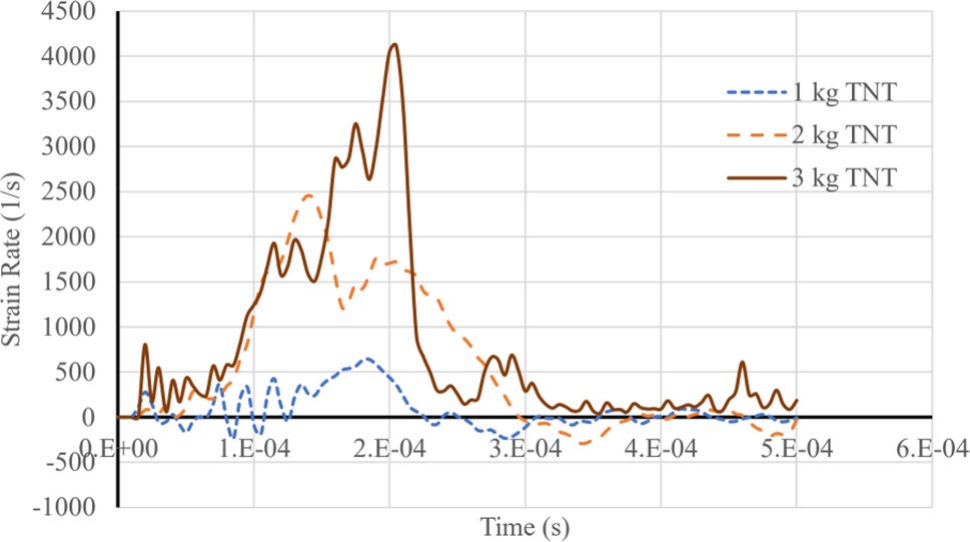
Strain rate of the top plate center position for three blast loads.

Besides the deformation velocity, the strain rates of the plates exhibit notably high magnitudes, necessitating the employment of a rate-dependent constitutive model in the analysis. Figure 13 illustrates the strain rate at the center of the top plate for three distinct loading scenarios. Upon evaluation, it is apparent that the strain rate falls within the order of thousands. Given these high strain rates, accurately modeling the material behavior requires the adoption of a rate-dependent material model. In this study, the widely-recognized Johnson–Cook model is employed to effectively capture the material response under the dynamic conditions induced by the blast loads. By utilizing such a model, the authors ensure a more comprehensive understanding of the structural response and performance under the high strain-rate blast loading conditions, thus enhancing the credibility and relevance of the findings derived from this investigation.

### Blast loading

In “Bio-inspired core design”, we concluded that the hybrid bionic design of the sandwich structure shows the best performance in absorbing the crushing energy among other candidates. There are multiple choices for blast modeling in the finite element softwares, including CEL, ALE, and ConWep methods. In the present study, the explosion phenomena is simulated using the ConWep method. The ConWep (Conventional Weapons Effects Program) method is a useful approach for modeling blast phenomena. This method is based on empirical relationships derived from experimental data for the interaction of shock waves with structures in the context of conventional weapons, such as explosions. The ConWep method enables users to simulate the effects of blast loading on structures without explicitly modeling the explosion and shock wave propagation. The ConWep method primarily focuses on the pressure–time history and blast load generated by the explosion, and it does not directly incorporate temperature changes associated with the explosion. The main purpose of ConWep is to provide an approximate method for simulating the effects of blast loading on structures, without explicitly modeling the explosion process and its associated phenomena, such as temperature changes, shock wave propagation, and chemical reactions. The Coupled Euler–Lagrange (CEL) method combines the Eulerian (fluid) and Lagrangian (solid) domains in a single simulation. This allows for the explicit modeling of the explosion, shock wave propagation, and their interaction with structures. CEL can accurately capture the effects of complex geometries and material interactions, making it well-suited for simulating blast events in a wide range of scenarios. In these advanced methods, you can model the explosive material and its behavior, including the detonation process, energy release, and temperature changes. This approach will provide more detailed and accurate results in terms of blast loading, temperature changes, and their effects on structures. Arbitrary Lagrangian–Eulerian (ALE) method is a hybrid approach that combines the advantages of both Lagrangian and Eulerian methods. It can be used to model complex fluid–structure interactions and large deformations, making it suitable for simulating blast events in various scenarios. The ALE method allows the mesh to move independently of the material, which can help avoid mesh distortion issues that may arise in purely Lagrangian or Eulerian methods. However, it’s essential to note that these methods are more computationally demanding compared to the ConWep method. Since in the GA-based optimization, a lot of simulations should be performed, computationally-efficient methods like the ConWep method are more appropriate to incorporate, as in the present study.

**Figure 14:**
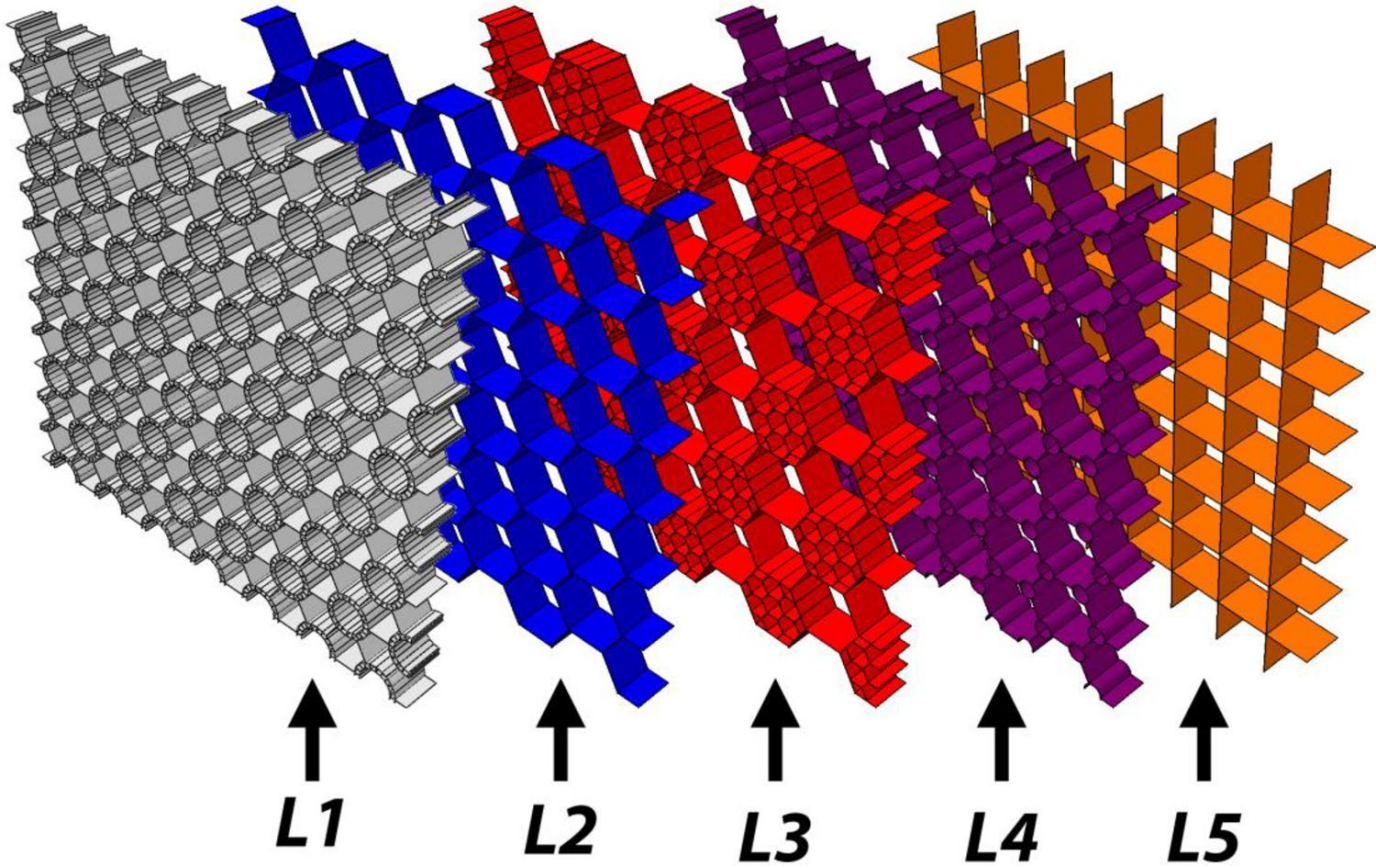
Sandwich core designs for blast load study.

**Figure 15:**
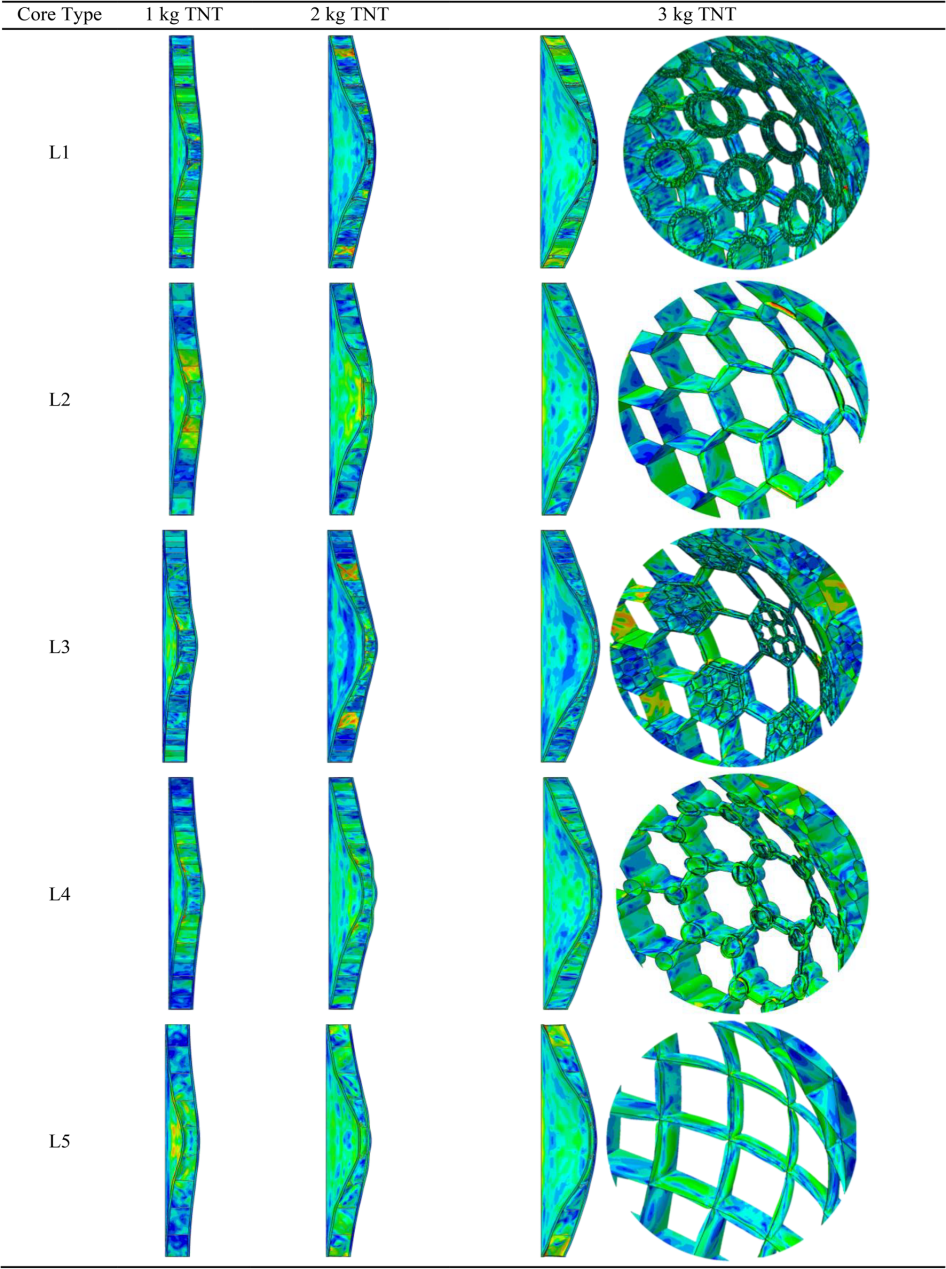
Deformed configuration of the sandwich structure and their core under blast loads.

Here, we examine this design under the blast loads and compare the results with the designs reported in similar studies by other researchers. These designs are designated by L1–L5, which are illustrated in Fig. 14. The L1 is the hybrid bionic design of the present study, L2 is the simple honeycomb structure, L3 is the hierarchical honeycomb inspired by pomelo peel which is presented by Zhang et al.^29^, L4 is the bionic design inspired by beetle's forewing which is presented by Chen et al.^31^, and L5 is the simple rectangular grid structure. All models have unit cells with equal-size peripheral circles. To eliminate the effects of the mass of the structures, all models have the same mass. The cores are placed between two 5 mm-thick plates. The blast load located at a 100 mm distance from the plate and three blast loads equivalent to 1, 2, and 3 kg TNT are considered in the analysis. The deformed configurations of the sandwich structures are depicted in Fig. 15.

The numerical results are listed in Table 10. It follows from these results that the proposed hybrid bionic design shows better results in absorbing the blast loads energy as well as the crushing energy, which is studied in the previous section. For the 1 and 2 kg TNT blasts, the present design is better than all other designs in both SEA and front/back plate deflections. For the 1 kg equivalent TNT blast, the present design shows 9.71 kJ/kg SEA, which is 13.4% more than the second-best design (the rectangular core). The SEA for the 2 kg TNT blast is 25.05 kJ/kg, 2% up from the L3 (hierarchical honeycomb), and for the 3 kg TNT, 41.01 kJ/kg, 1.4% less than the L3 design.

**Table 10:** The results of the blast loading on different core designs.

Core design	Wall thickness (mm)	TNT (kg)	SEA (kJ/kg)	Front plate max deflection (mm)	Back plate max deflection (mm)	Mass (kg)
L1	0.7	1	9.71	42.24	24.23	8.76
		2	25.05	93.68	59.44	8.76
		3	41.01	130.2	84.87	8.76
L2	2.51	1	8.11	45.97	29.81	8.76
		2	22.04	82.74	58.14	8.76
		3	35.80	120.6	82.46	8.76
L3	1.305	1	8.17	35.34	28.39	8.76
		2	24.58	88.70	65.54	8.76
		3	41.62	130.4	88.02	8.76
L4	1.691	1	8.55	42.49	30.31	8.76
		2	22.85	83.92	61.09	8.76
		3	36.72	120.8	86.84	8.76
L5	2.242	1	8.56	43.96	25.99	8.76
		2	23.39	81.10	52.64	8.76
		3	36.36	121.3	80.09	8.76

## Conclusions

For the sandwich structures' central component, a hybrid bionic design is suggested in order to absorb the blast loads' energy. The core is made up of the tubular reinforcers that have been added to the grid structure, which were inspired by the design of the forewing of a beetle. Different grid structures are tested under crushing loads to determine the optimal scenario, which is the triangle grid, in order to create the structure with the optimum performance. Instead of using a simple tube, we presented a bio-inspired tubular structure made of two co-axial tubes joined by radial ribs (inspired by the structure of bamboo). The structure is investigated under three blast loads comparable to 1, 2, and 3 kg TNT after the design parameters have been optimized and the results compared with those presented in the literature. The following remarks could be made.

- It is advised that the mass be kept constant in optimization or comparative studies because the mass of the structures impacts their energy absorption characteristics (including the SEA, which is the absorbed energy per unit mass).
- The triangular grid gives better results than the conventional honeycomb structure, either the pure grid or reinforced with some stiffeners.
- For tubular stiffeners, it is the best practice to replace them with the bionic bi-tubular design proposed in this study.
- The hybrid bionic design presented here is better than the bio-inspired design of Ref.^31^, which is a honeycomb reinforced with tubular stiffeners inspired by the beetle's forewing structure.
- The difference between blast loads and crushing loads is that the former does not yield uniformly deformed structures. Therefore, the sub-structure located at the blast's epicenter is crucial to the overall features of the structure's ability to absorb blast energy. Since the location of the blast is typically unknown, their behavior may differ from what was intended. We placed the blast point in front of the sub-structure in order to acquire the best state of that design and compare it with the provided design. The hierarchical design of Ref.^26^ consisted of a smaller honeycomb grid in some of the larger honeycomb cells (case L3 in Fig. 10). The results show that for the 1 and 2 kg equivalent TNT blasts, the proposed design shows better energy absorption characteristics than the previous design.

## Data Availability

The datasets used and/or analyzed during the current study available from the corresponding author on reasonable request.
